# Research on the Fracture Behavior of Steel-Fiber-Reinforced High-Strength Concrete

**DOI:** 10.3390/ma15010135

**Published:** 2021-12-24

**Authors:** Shanming Qin, Danying Gao, Zhanqiao Wang, Haitang Zhu

**Affiliations:** 1 School of Water Conservancy Engineering, Zhengzhou University, Zhengzhou 450001, China; qinshanming0210@163.com (S.Q.); gdy@zzu.edu.cn (D.G.); htzhu@zzu.edu.cn (H.Z.); 2 College of Civil Engineering, Henan University of Engineering, Zhengzhou 451191, China

**Keywords:** steel fiber reinforced high-strength concrete, fracture toughness, fracture energy, three-point bending test

## Abstract

The behavior of steel fiber concrete, which is the most widely used building material, has been widely examined. However, methods for calculating Fracture parameters differ by fracture behavior of SFHSC with different strengths. In this study, the fracture behavior of steel-fiber-reinforced high-strength concrete (SFHSC) was -investigated using three-point bending tests. A total of 144 notched concrete beams with a size of 100 mm × 100 mm × 515 mm were tested for three-point bending in 26 groups. The effects of the steel fiber volume ratio, steel fiber type, and relative notch depth on the fracture toughness (K_IC_) and fracture energy (G_F_) of SFHSC specimens were studied. The results show that an increase in the volume fraction of steel fiber (*ρ_f_*) added to high-strength concrete (HSC) significantly improves the fracture behavior of HSC. As compared to milled and sheared corrugated steel fibers, cut bow steel fibers significantly improve the fracture behavior of SFHSC. The effect of incision depth changes on the K_IC_ and G_F_ of SFHSC and HSC for the comparison group has no common characteristics. With an increase in incision depth, the values of K_IC_ of the SFHSC specimens decrease slightly. The G_F0.5_/G_F0.4_ of the SFHSC specimens show a decreasing trend with an increase in *ρ_f_.* According to the test results, we propose calculation models for the fracture behavior of SFHSC with different strengths. Thus, we present a convenient and accurate method to calculate fracture parameters, which lays a foundation for subsequent research.

## 1. Introduction

With the development of modern building technology, numerous varied complex structures have emerged with cement concrete performance requirements; therefore, methods for improving the performance of concrete are always of interest to researchers [1,2]. Currently, there is an increasing demand for high-strength or even ultrahigh-strength concrete, with low cost and few construction difficulties [3]. High-strength concrete (HSC) is a typical representative of concrete in the high-tech era; however, it is more brittle than ordinary concrete, and damage often occurs suddenly and without warning. Undoubtedly, many unsafe factors are added to large and complex concrete structures [4,5].

To enhance the strength of the concrete matrix and to reduce the toughness, investigators have trialed different treatments. Studies have found that merging fibers into concrete can enhance the tensile strength, fracture toughness, dynamic mechanical properties, and the durability of concrete [6,7,8]. Fiber types such as carbon fiber, polypropylene, glass, and steel fiber are commonly used to enhance the strength of the concrete matrix [9,10,11]. Among the various suggested approaches, the implementation of steel fibers is one of the most widely exploited methods to improve the concrete mechanical behavior because it is easily available and contained within the concrete mix [12]. The most advantageous element of steel fiber is that it increases the ductility of concrete after microcracks are formed [13,14,15,16,17,18]. The addition of steel fiber to concrete changes the strain capacity and reduces the brittleness of HSC [19]. A study by Yoo et al. [20] showed that steel fiber contents of over 1.0% could significantly improve the post-peak ductility and compressive strength of concrete. Şemsi et al. [21] reached a similar conclusion. Therefore, in order to improve the mechanical properties of HSC and increase the cracking strength, a specific amount of steel fiber is usually added to the HSC matrix. SFHSC has excellent mechanical properties and can be used in large-span, high-rise, heavy-load, and thin-walled structures. If SFHSC is used in structures that bear dynamic loads, the effect will be more significant.

Many studies [13] have used concrete-notched beams to explore the crack growth of concrete beam specimens. The fracture properties of conventional cracked concretes are commonly related to the relative notch depth [22]. If the notch changes the net section strength (σ_net_) of the material, then the material can be considered to have notch sensitivity [23]. Therefore, notch sensitivity means that the value of σ_net_ may be reduced due to the existence of notches. Commonly, materials without notch sensitivity can be directly analyzed using classical mechanics. Notch-sensitive materials must be analyzed using the principles of fracture mechanics. There is a microcrack zone at the tip of a concrete crack. It is generally believed that a metal elastoplastic fracture mechanics model such as the J-integral model is not suitable for concrete. Hillerborg proposed a fictitious crack model (FCM) [24,25] that eliminated the influence of metal fracture mechanics conceptually, and created a new idea for the study of concrete nonlinear fractures. Hardened cement mortar is generally considered to be a notch sensitive material. Hu, Y. and Wang, J. et al. [26,27] showed that steel fiber concrete displayed notch sensitivity. In this study, we studied the notch sensitivity of SFHSC using three-point bending tests of notched beams. By adding steel fiber to the concrete matrix, the strength and toughness of concrete will be improved, especially for high-strength concrete [28].

A large number of studies have shown that a variety of fibers can enhance the fracture properties of concrete. Zinnur Çelik et al. [29,30] revealed that polypropylene-fiber-reinforced concrete had high impact resistance and fracture energy. Zongcai Deng [31] investigated the fracture and fatigue properties of carbon-fiber-reinforced concrete (CFRC) through three-point bending tests. The results proved that the threshold of the fracture parameters and the ability to resist the bending fatigue load were greater for CFRC than for ordinary concrete. However, the cost of carbon, polypropylene, and other fibers compared to steel fibers is too high for use in practical engineering.

Since it has been proven that many factors affect the mechanical properties and fracture behavior of concrete, research and testing should be implemented for Steel fiber parameters, test methods and dimensional variables of test pieces, etc. [32,33,34,35,36] Kazemi et al. [37] studied the fracture behavior of steel fiber reinforced high-strength concrete (SFRHSC), and used the work of fracture method (WFM) and the size effect method (SEM) to measure the fracture parameters. The results showed that on enhancing the content of steel fibers, the fracture energy in WFM and SEM increased; however, the increment in WFM was more significant, with HSC becoming more ductile for introducing the fibers. Zhang et al. [38] used a servo hydraulic press and a drop-hammer impact device to perform the three-point bending test on a steel fiber concrete (SFRC) notched beam. As the loading rate increased, the fracture energy and peak load were observed to increase significantly. However, SFHSC with different strengths may be used in designing large structures. Based on the reinforcement mechanism theory, of steel fiber reinforced concrete and in consideration of the influence of crack subcritical propagation on fracture behaviors, we establish some parameter calculation models of SFHSC suitable for different strengths, that are conducive to the calculation and analysis of large structures. Therefore, it is essential to systematically explore the fracture performance of SFHSC, so as to identify more convenient and accurate calculation methods.

The test is mainly aimed at examining the influence of the steel fiber volume ratio, steel fiber type, relative notch depth, and other factors on the fracture toughness (K_IC_) and fracture energy (G_F_) of SFHSC. To this end, rational calculation models for the fracture behaviors of SFHSC are developed.

## 2. Materials and Methods

### 2.1. Material

**Figure 1:**
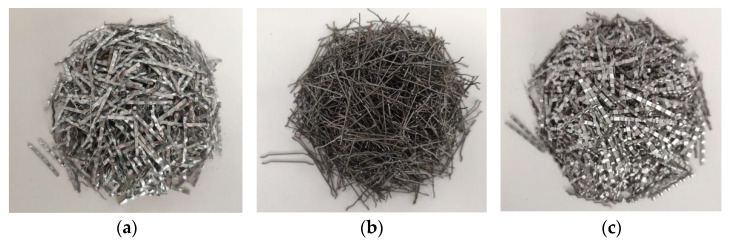
Sketch of the specimen shape and dimension. (**a**) Milled steel fiber; (**b**) Bow steel fiber; (**c**) Shear corrugated steel fiber.

In the test mixture, the fundamental material, PO 42.5 ordinary Portland cement, with a 28-day strength greater than 42.5 was used. The standard water/cement ratio of SFHSC was 0.3, and the sand ratio was 0.34. The coarse aggregate was composed of crushed limestone with a maximum particle size of 20 mm. The fine aggregate was medium coarse river sand with a fineness modulus of 3.39. According to the water/cement ratio of the SHFSC, in this test, a 1% FDN-1 superplasticizer with a strong dispersing effect on cement was mixed with cement through daily drinking water. The steel fiber types were milled steel fiber (MF), bow steel fiber (BF), and shear corrugated steel fiber (SF). The types of steel fiber are shown in Figure 1. The material properties of the steel fiber types are shown in Table 1.

### 2.2. Mix Design

The test was designed according to the test method of the China Engineering Construction Standardization Association [39,40]. The base concrete mix ratio was water/cement/fine aggregate/coarse aggregate/superplasticizer = 146:487:618:1199:4.87. The SFHSC increased water consumption by 10 kg from the reference group concrete to the fiber group concrete. According to the requirements of the specification [39,40], SFHSC increased water consumption by 10 kg from the reference group concrete to the fiber group concrete. For every 0.5% increment in *ρ_f_*, the water consumption increased by 8 kg. Six types of samples with 0%, 0.5%, 1.0%, 1.5%, 2.0%, and 2.5% steel fibers in the *ρ_f_* were prepared.

In order to eliminate the influence of the strength variation of the base concrete on the fracture behavior test results of the SFHSC, ordinary HSC with the same mix ratio but without steel fiber was used as the comparison test specimen. The mix proportions of SFHSC and HSC are shown in Table 2.

### 2.3. Specimen Preparation

**Figure 2:**
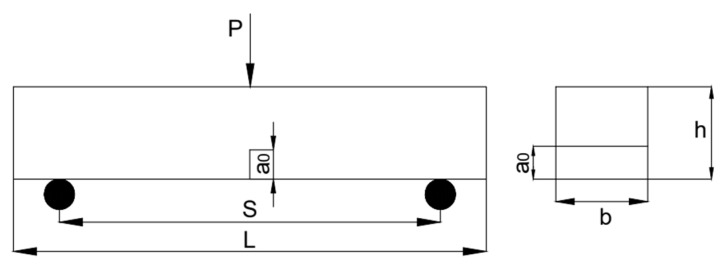
Sketch of the specimen shape and dimension.

An SFHSC notch beam specimen is shown in Figure 2, in which the specimen was designed as a cuboid of 100 mm× 100 mm × 515 mm, and the beam span (S) was 400 mm. The notch was located at the side of the pouring face of the test piece. The incision was made using a high-speed concrete cutting machine, and the width of the saw gap was around 3 mm. Furthermore, the cut depths a_0_ were 40 mm and 50 mm. The relative notch depth of the prefabricated cracks of the specimen included a_0_/h = 0.4 and 0.5. The SFHSC design-strength grade was FC50.

**Figure 3:**
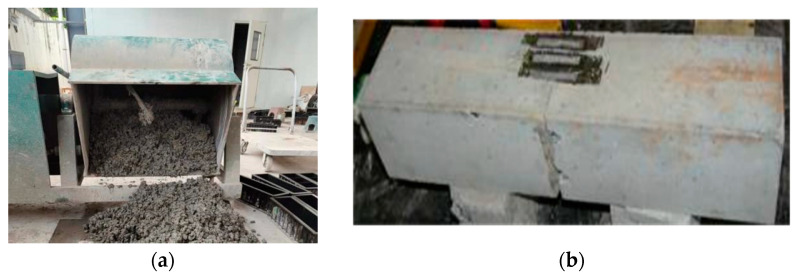
Mixed concrete (**a**) and processed SFHSC specimen (**b**).

The test materials were appropriately cleaned by removing the impurities before fabricating the SFHSC specimens. The SFHSC specimens were formed using a mixer to mix the concrete, and a vibration table was used for forming. The specimens were cured for 28 days at a temperature of 20 ± 2 °C and a relative humidity of no less than 95%. The test age was 60–70 d. The mixed concrete and processed SFHSC specimens are shown in Figure 3a,b.

### 2.4. Test Method

#### 2.4.1. Three-Point Bending Test Procedure

**Figure 4:**
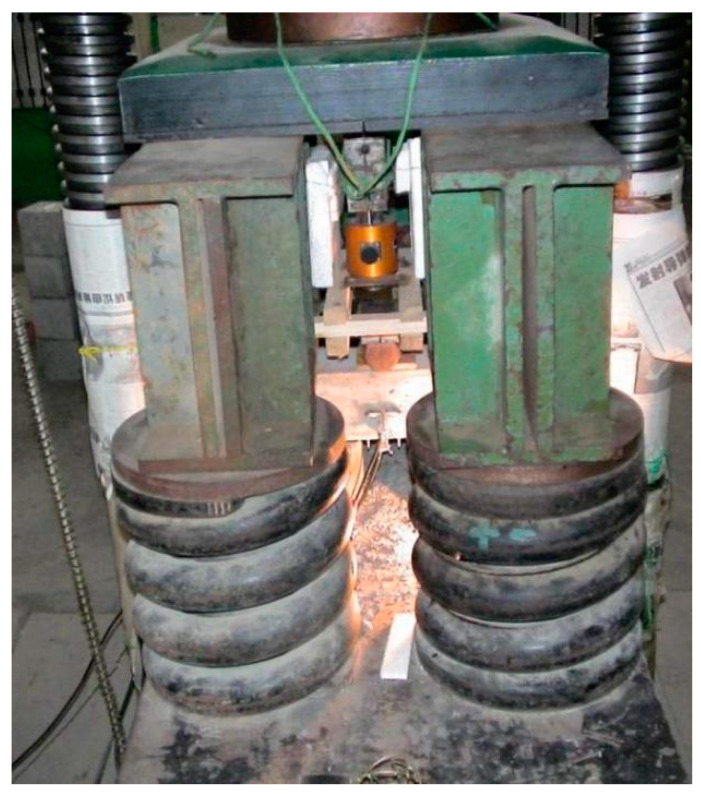
The photo of three-point bending test machine.

**Figure 5:**
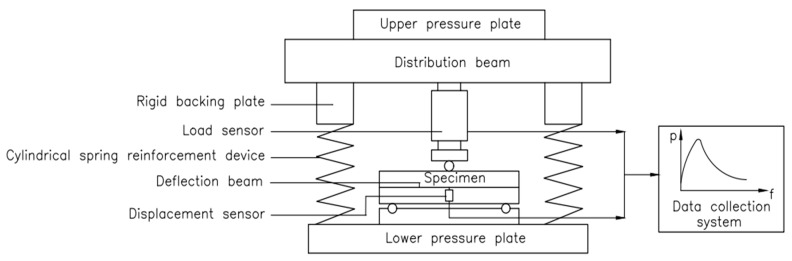
The schematic diagram of the TPBTs loading device.

The three-point bending tests (TPBTs) were carried out on a 2000 kN universal testing machine (Figure 4). In the test, cylindrical springs with greater stiffness were used to reinforce the test machine and ensure that any additional deformation energy generated by the sudden unloading of the testing machine was absorbed at the moment of failure of the test specimen; therefore, the test process was reliable and stable. During the test, the beams were arranged above the two supporting points. The load was applied downwards, directly above the midpoint of the two support points, and measured by a load sensor in the range of 0 to 30 kN. In addition, an extensometer was used on the lower surface of the beam to obtain the crack opening displacement (CMOD). The test adopted a continuous loading method, and used a computer data collection system to realize automatic data collection. The collection rate used different collection frequencies according to the type of component. Among these, the value of the HSC component was 2 times/s, whereas the SFHSC component had a value of 1 time/s. At the same time, the load CMOD (P-CMOD) and load deflection (P-δ) were analyzed. According to the curve, and combined with the observation of the test phenomena in the test process, the fracture behavior of SHFSC was qualitatively and quantitatively analyzed. The schematic diagram of the TPBTs loading device is shown in Figure 5.

#### 2.4.2. Test Parameter Calculation

In this test, the fracture toughness was calculated using ASTM Formulas (1) and (2) as follows:

(1) $$K_{IC}=\frac{P_{\max}S}{th^{3/2}}f\left(\frac{a_{e}}{h}\right)$$

(2) $$f\left(\frac{a_{e}}{h}\right)=2.9{\left(\frac{a_{e}}{h}\right)}^{\frac{1}{2}}-4.6{\left(\frac{a_{e}}{h}\right)}^{\frac{3}{2}}+21.8{\left(\frac{a_{e}}{h}\right)}^{\frac{5}{2}}-37.6{\left(\frac{a_{e}}{h}\right)}^{\frac{7}{2}}+38.7{\left(\frac{a_{e}}{h}\right)}^{\frac{9}{2}}$$

where *P*_max_ is the peak load of the three-point bending beam, *S* is the span between the three-point bending beam supports, *b* is the thickness of the test specimen, and *a*_e_ is the equivalent crack length.

Its value is an iterative calculation according to the F_max_ and *CMOD_C_* obtained from the p-*CMOD* curve measured in the test, and it is calculated according to Equation (3) as follows:

(3) $$CMOD_{C}=\frac{6P_{\mathrm{mas}\;}Sa_{e}}{th^{2}E}f_{1}\left(\frac{a_{e}}{h}\right)$$

(4) $$f_{1}\left(\frac{a_{e}}{h}\right)=1.45-2.18\frac{a_{e}}{h}+13.71{\left(\frac{a_{e}}{h}\right)}^{2}-5.96{\left(\frac{a_{e}}{h}\right)}^{3}-36.9{\left(\frac{a_{e}}{h}\right)}^{4}+70.7{\left(\frac{a_{e}}{h}\right)}^{5}$$

where *E* is the elastic modulus, and its value is the measured value of the concrete elastic modulus of SFHSC, and the comparison group obtained in the experiment; *CMOD_C_* is the critical crack mouth opening displacement.

The calculation Formula (5) of the fracture energy *G_F_* is [25] as follows:

(5) $$G_{F}=\frac{W}{A_{lig}}=\frac{W_{0}+\left(gS+2P_{1}\right)\delta_{0}}{b\left(h-a_{0}\right)}$$

where *A*_lig_ is the area of the fractured ligament perpendicular to the direction of tensile stress, *g* is the linear distributed load of the beam’s dead weight, *a*_0_ is the length of the prefabricated crack of the test specimen, and *δ*_0_ is the ultimate deflection of the three-point bending beam.

## 3. Results and Discussions

The three-point bending test results for the specimens are shown in Table 3.

### 3.1. Fracture Toughness

#### 3.1.1. The Effect of *ρ_f_* on the Fracture Toughness of SFHSC

**Figure 6:**
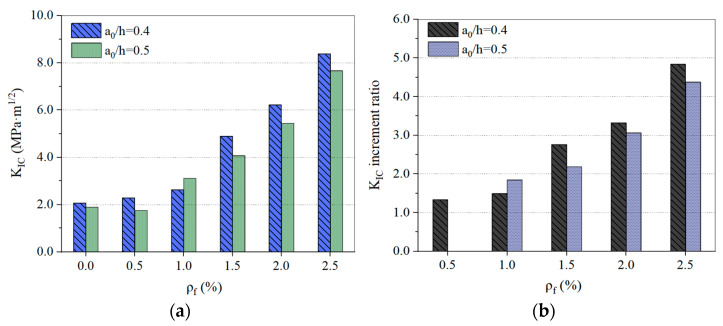
Influence of *ρ_f_* on the K_IC_ (**a**) and K_IC_ increment ratio (**b**) of SFHSC.

The influence of *ρ_f_* on the K_IC_ of the SHFSC specimens under the conditions of the relative depth of the two notches is shown in Figure 6a. In addition to the SFHSC specimens with *ρ_f_* = 0.5% and a_0_/h = 0.5, the K_IC_ of SFHSC was improved to different degrees compared to HSC. The K_IC_ values of the SFHSC specimens show a good increasing trend with an increase in *ρ_f_*. For every 0.5% increment in *ρ_f_*, the average increase in the K_IC_ value were as follows: when a_0_/h = 0.4, K_IC_ increases by 40.7%; when a_0_/h = 0.5, K_IC_ increases by 45.7%. According to the results, when *ρ_f_* reaches a certain value, the addition of steel fiber can increase the K_IC_ of SFHSC. Kazemi et al. [37] also obtained similar results.

Figure 6b shows the effect of *ρ_f_* on the K_IC_ increment ratio of the SFHSC specimens. It can be seen that there are no test data for K_IC_ due to the *ρ_f_*= 0.5% and a_0_/h = 0.5 comparison group specimen variation; all the other K_IC_ increment ratio values are greater than 1. Moreover, the value of the K_IC_ increment ratio also increases to varying degrees as *ρ_f_* increases. Regarding the variation range of the increment ratio, when a_0_/h = 0.4, the increment ratio values are between 1.33 and 4.84, and the average increment ratio value is 2.75. When a_0_/h = 0.5, the increment ratio values are between 1.85 and 4.38, and the average increment ratio value is 2.87.

**Figure 7:**
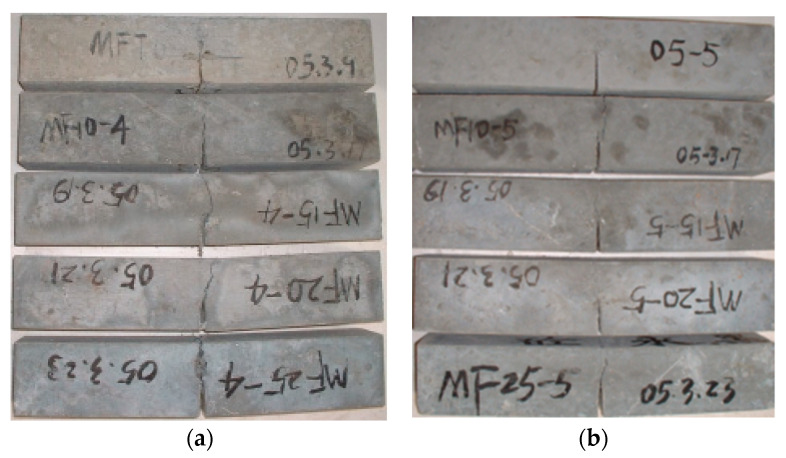
Comparison of crack face failure mode between SFHSC beams reinforced with different *ρ_f_* at a_0_/h = 0.4 (**a**) and a_0_/h = 0.5 (**b**).

Figure 7a,b demonstrates the comparison of the failure modes of the SFHSC specimens with *ρ_f_* for two relative notch depths. The crack propagation of the SFHSC specimen is noted to be tortuous, and the crack exhibits multi-point cracking. Moreover, on increasing the fiber volume fraction, the crack propagation of the SFHSC specimen becomes more complex.

**Figure 8:**
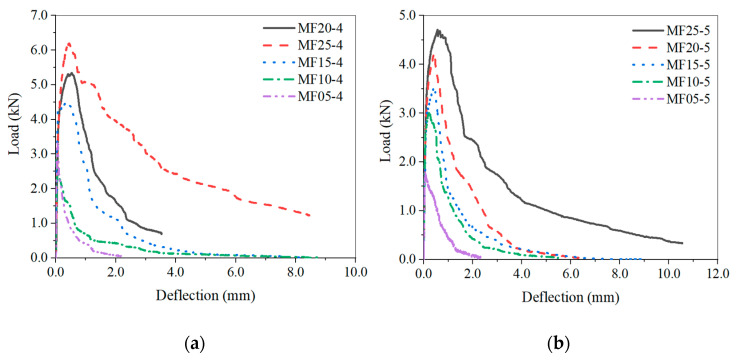
Typical load deflection curves of the SFHSC beams with a_0_/h = 0.4 (**a**) and a_0_/h = 0.5 (**b**).

A comparison of typical load deflection (P-δ) curves of SFHSC specimens with different *ρ_f_* under the conditions of two relative opening depths is shown in Figure 8a,b. The peak loads of the curves in the figures from bottom to top are MF05, MF10, MF15, MF20, and MF25. It can be observed that before the initial crack of the SHFSC specimen, the load increases with an increase in the deformation, and *ρ_f_* has no significant effect on the curve. As the load continues to increase, the microscopic cracks appear in the concrete matrix. The cracks continue to grow until the appearance of macro cracks, and the SHFSC specimen is in the elastoplastic stage. In the process of gradual pull-out, the steel fiber connects the concrete across the crack. It mainly controls the propagation of cracks in the concrete matrix and limits the crack width. After the concrete matrix cracks, the cracks are bridged [41,42]. With an increase in *ρ_f_*, the number of fibers across the crack increases correspondingly, and the ability to transfer stress is also relatively improved. If the nonlinear development stage is longer, the development of microcracks in the fracture process area is found to be relatively sufficient, and the absorbed external load energy increases accordingly. It suggests that the fracture performance can be enhanced with an increase of the peak load and the ultimate deflection.

#### 3.1.2. Effect of Steel Fiber Type on Fracture Toughness of SFHSC

**Figure 9:**
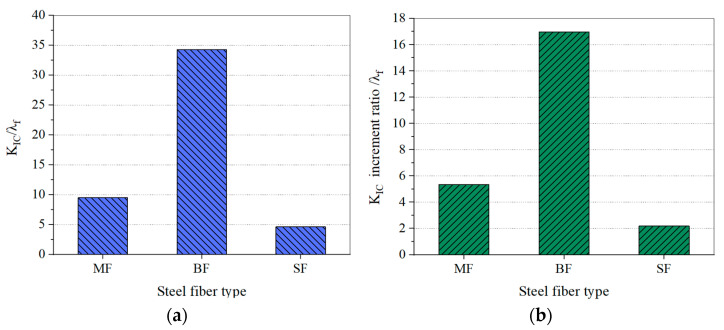
Influence of steel fiber types on the K_IC_ (**a**) and K_IC_ increment ratio (**b**) of SFHSC.

Due to the different effects of steel fiber types and steel fiber lengths on the fracture behavior of SFHSC specimens we introduce, in this study, the concept of characteristic fracture parameters, defined as the SFHSC fracture parameter/characteristic content of steel fiber. The characteristic K_IC_ and increment ratio of the same HSC matrix and different steel fiber types of SFHSC specimens are shown in Figure 9a,b when *ρ_f_* = 1.5% and a_0_/h = 0.4. It indicates that the BF improves the K_IC_ most significantly, followed by the MF. The characteristic K_IC_ values of the BF are 3.62 and 7.41 times those of the MF and SF, respectively, and the K_IC_ increment ratios are 3.17 and 7.77 times those of the MF and SF, respectively.

Similar to the results of this study, those by X W Wang et al. [43] show that the types of steel fiber deliver a greater impact on the fracture toughness of Steel-Fiber-Reinforced Concrete. The test results demonstrate that Bekaert-type fiber has a better performance in improving the fracture toughness.

**Figure 10:**
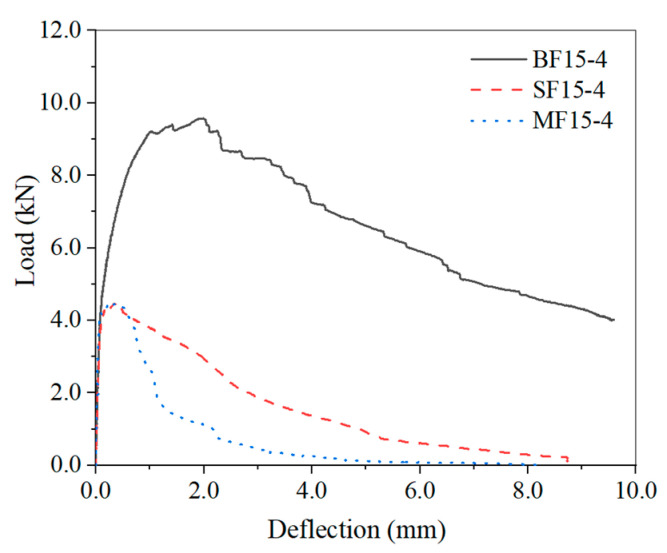
Typical load-deflection curves of SFHSC beam reinforced.

The typical load deflection curves of SFHSC for different steel fiber types are shown in Figure 10. In the early stage of loading, due to the synergistic effect of the steel fiber and matrix concrete, the load–deflection curves of SFHSC specimens with different steel fiber types are not found to be significantly different. As the load increases, cracks appear in the concrete matrix. Owing to the different steel fiber types, the load–deflection curves are different. The BF and SF have a greater increment effect on high-strength concrete than MF. However, the BF high-strength concrete exhibits a weak stress-strengthening phenomenon in the tests, and exhibits metal stress–strain characteristics to some extent. In addition, the occurrence of the second peak may be related to the spalling of large aggregates, and the reinforcement of the BF should be the main reason for the stress strengthening. Although the process requires a certain amount of energy, it also leads to the release of stress and strain at the fracture area, which weakens the toughening effect of the fiber. In general, the width of the zone is related to the half-length of the fiber length and the maximum particle size of the aggregate used. The peak load and ultimate deflection of SFHSC can be significantly increased by the BF, and the bearing capacity of SFHSC specimens after the peak can be significantly increased by the end hook of the BF. Test curve of BF15-4 is steeper and the descent section is gentler. Due to the significant deformation of the BF high-strength concrete beam, even if the deflection test dial indicator is adjusted during the loading process, it is still difficult to measure its ultimate deflection. However, this trend is sufficient to show that BF has significant advantages over other types of steel fibers for improving HSC fracture behavior. There is no significant difference in peak load performance between MF and sheared SF, but from the descending section of the typical test curve, it can be found that the toughening effect of SF is slightly better than that of MF.

#### 3.1.3. The Influence of Incision Depth on Fracture Toughness of SFHSC

**Figure 11:**
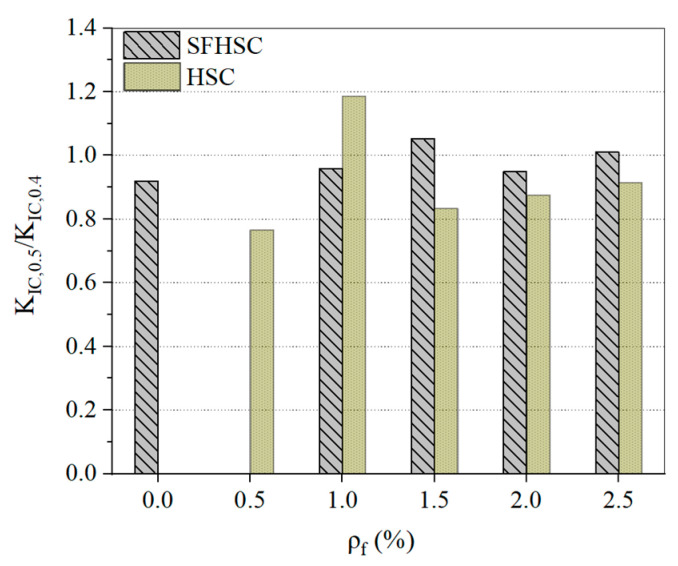
Influence of notch depth on the K_IC_ of SFHSC and HSC.

Figure 11 reveals the comparison of the K_IC_ ratios of the milled SFHSC, i.e., the comparison group and the reference group HSC specimens under the conditions of different *ρ_f_* corresponding mix ratios (K_IC0.5_/K_IC0.4_). It shows that the K_IC0.5_/K_IC0.4_ values of the SFHSC specimens are all less than 1, and the average value is 0.92, except for *ρ_f_* = 1.0%, which is the variation specimen. The value of K_IC_ has a decreasing trend with an increase in the notch depth. With an increase in *ρ_f_*, the value of K _IC0.5_/K_IC0.4_ has an increasing trend. The fracture toughness notch sensitivity gradually decreases. The value of K_IC0.5_/K_IC0.4_ fluctuates irregularly between 0.92 and 1.05 for the HSC specimens of the reference group and the comparison group of SFHSC. Its average value is 0.98. According to the test data in this study, it can be inferred that SFHSC materials are notch sensitive, while HSC materials are notch insensitive.

### 3.2. Fracture Energy

#### 3.2.1. The Effect of *ρ_f_* on the Fracture Energy of SFHSC

**Figure 12:**
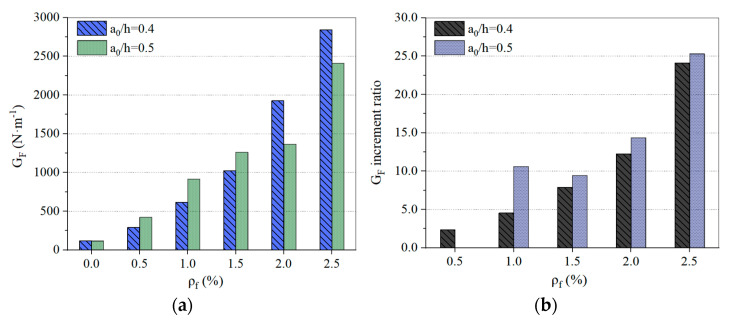
Influence of *ρ_f_* on the G_F_ (**a**) and G_F_ increment ratio (**b**) of SFHSC.

The fracture energy of concrete is one of the important parameters for understanding concrete properties and determining the design standards of large concrete structures [15]. Figure 12a,b shows the effect of *ρ_f_* on the G_F_ and G_F_ increment ratio for SFHSC under different relative notch depth conditions. The results presented in Figure 12a reveal that no matter the depth of the incision changes, the addition of steel fiber can significantly increase G_F_ compared with the reference HSC. Figure 12a shows that for every 0.5% increase in *ρ_f_*, the average growth rate increases; when a_0_/h = 0.4, the G_F_ increases by 78.39%, and when a_0_/h = 0.5, G_F_ increases by 59.92%. As shown in Figure 12b, the G_F_ increment ratio is greater than 1 with the addition of steel fiber. The G_F_ increment ratio also shows a good increasing trend with an increase in *ρ_f_*; especially, the increase in SFHSC with high fiber volume content is more significant. The change in the G_F_ increment ratio for the SFHSC specimens is that, when a_0_/h = 0.4, the increment ratio values are between 2.36 and 24.08. When a_0_/h = 0.5, the increment ratio values are between 9.42 and 25.31. In Figure 12b, the increment ratio values of the specimens with *ρ_f_* = 1.0% and a_0_/h = 0.5 are clearly larger, which is due to the slight local damage caused by the HSC specimens during demolding. Therefore, the G_F_ value of the HSC specimens in the comparison group is only 86.22 N·m^−1^.

By comparing the test data with other relevant tests [16,37], it can be concluded that despite the differences in concrete mixtures, sample geometry and size between different studies, the fracture energy is greatly affected by the fiber volume ratio of SFHSC.

#### 3.2.2. Effect of Steel Fiber Type on the Fracture Energy of SFHSC

**Figure 13:**
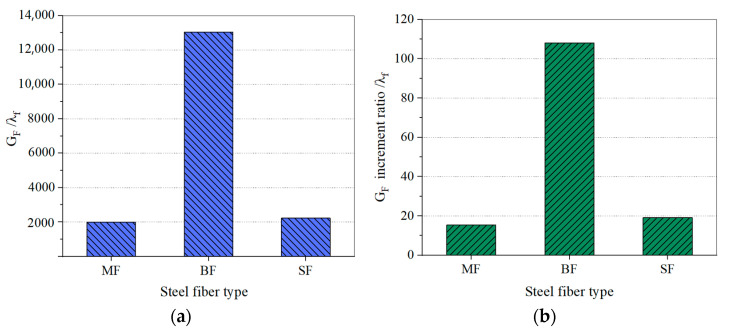
Influence of Steel Fiber Types on the G_F_ (**a**) and G_F_ Increment Ratio (**b**) of SFHSC.

For the specimens with the same pouring conditions and *ρ_f_* = 1.5%, the effects of different steel fiber types on the G_F_ and the G_F_ increment ratio of HSC are shown in Figure 13a,b. Regardless of the G_F_ or the G_F_ increment ratio, BF plays a significant role in the steel fiber, showing an excellent fiber-reinforced effect. The characteristic G_F_ of BF is 6.57 and 5.85 times that of MF and SF, respectively, and its characteristic G_F_ increment ratio is 7.06 and 5.67 times that of MF and SF, respectively.

#### 3.2.3. The Influence of Incision Depth on Fracture Energy of SFHSC

**Figure 14:**
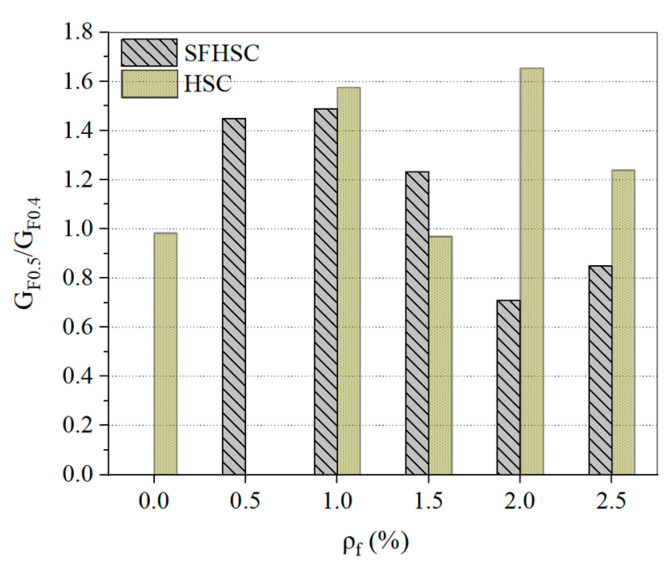
Influence of notch depth of beam on the fracture energy of SFHSC.

The fracture energy ratio values of the specimens at a_0_/h = 0.5 and a_0_/h = 0.4 under different *ρ_f_* conditions (G_F0.5_/G_F0.4_) are shown in Figure 14. According to the results in the figure, the G_F0.5_/G_F0.4_ values of the SFHSC specimens show a decreasing trend with an increase in *ρ_f_*. The maximum and minimum values are 1.48 and 0.71, respectively. The G_F0.5_/G_F0.4_ is greater than 1 when *ρ_f_* is less than 1.5%, and when *ρ_f_* is greater than 1.5%, the G_F0.5_/G_F0.4_ is less than 1. A notch strengthening phenomenon was observed for SFHSC with an increase in *ρ_f_*. However, the changes in the values of G_F0.5_/G_F0.4_ of HSC specimens under different *ρ_f_* conditions have no obvious regularity, which means that a change in *ρ_f_* has no effect on the G_F0.5_/G_F0.4._ There are few studies that investigate the effect of notch depth changes on the G_F_ of SFHSC; therefore, it is not possible to make a further horizontal comparison, and further tests are needed.

### 3.3. Unified Calculation Model of SFHSC Behavior

The K_IC_ calculation model of SFHSC is defined as (6)

(6) $$K_{\mathrm{sIC}}=\left(1+\alpha_{sf,k}\lambda_{f}^{2}\right)K_{\mathrm{IC}}$$

where *K*_sIC_ is the fracture toughness of SFHSC, *K*_IC_ is the fracture toughness of the comparative group of concrete, *λ*_f_ is the characteristic parameter of steel fiber content, and *α_sf,k_* is the increment coefficient of steel fiber to concrete fracture toughness considering the effect of steel fiber spacing.

**Figure 15:**
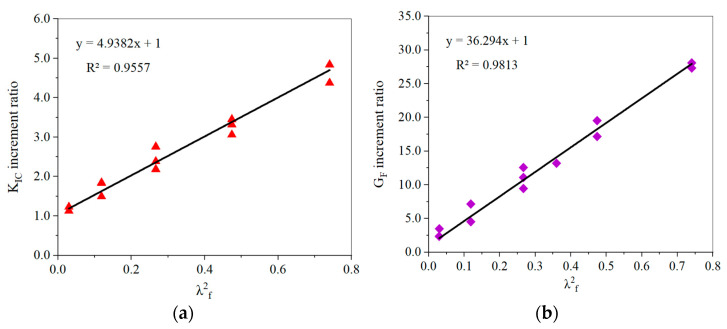
Regression curve of the K_IC_ increment ratio v. s. *λ*^2^_f_ (**a**) and G_F_ increment ratio v (**b**).

According to the SFHSC fracture toughness test, the *K*_IC_ increment coefficient of steel fiber to concrete, considering the fiber spacing effect, is *α*_sf,k_ = 4.9382. The average value of the ratio of the *K*_IC_ measured value to the calculated value according to Formula (6) is 1.0581, the mean square error is 0.1125, and the coefficient of variation is 0.1063. Figure 15a shows the regression relationship between the measured *K*_s__IC_ increment ratio and *λ*_f_^2^.

The calculation model of the *G_F_* of SFHSC is defined as (7):

(7) $$G_{sF}=\left(1+\alpha_{sf,g}\lambda_{f}^{2}\right)G_{F}$$

where *G*_sF_ is the fracture energy of SFHSC, *G_F_* is the fracture energy of the comparative group of concrete, and *α*_sf,g_ is the increment coefficient of MF to concrete fracture energy considering the effect of steel fiber spacing.

According to the SFHSC fracture energy test, the increment coefficient of the fracture energy of steel fiber concrete considering the effect of fiber spacing is *α*_sf,g_ = 36.296. Excluding the significant variation data of MFT10-5, the average value of the ratio for the measured value of steel-fiber-reinforced concrete *G_F_* to the calculated value according to Formula (7) is 1.0031, the mean-square-error is 0.1054, and the coefficient of variation is 0.1051. Figure 15b shows the regression curve of the experimental *G_SF_* increment ratio and λ_f_^2^.

## 4. Conclusions

Based on the TPBT of the notched concrete beam, the influence of the type of steel fiber, the volume fraction of steel fiber, and the relative notch depth on the fracture behavior of SHFSC was studied. In actual large-scale structural design projects, the fracture performance of the selected SHFSC can be measured and judged based on the conclusions and formulas offered in this study. The main conclusions are as follows:

(1) Steel fiber can significantly increase the K_IC_ value of HSC. The *ρ_f_* increases by 0.5%. When a/h_0_ = 0.4 and 0.5, K_IC_ increases by 40.7% and 45.7%, respectively, and the average increment ratios are 2.75 and 2.87, respectively.
(2) Steel fiber can more effectively improve the G_F_ of HSC. The addition of steel fiber can significantly increase the HSC fracture energy, and leads to a steeper load–deflection curve ]. The peak load, peak deflection, and ultimate deflection all increase to varying degrees with an increase in *ρ_f__._*
(3) The BF is most effective for improving the fracture behavior of SHFSC. The K_IC_ values of the BF are 3.62 and 7.41 times those of the MF and SF, respectively. The K_IC_ increment ratios of the BF are 3.17 and 7.77 times those of the MF and SF, respectively. The G_F_ values of the BF are 6.57 and 5.85 times those of the MF and SF, respectively. The G_F_ increment ratios of the BF are 7.06 and 5.67 times those of the MF and SF, respectively.
(4) A change in notch depth has no significant effect on the K_IC_ of HSC specimens. The K_IC_ of SFHSC specimens decreases slightly with an increase in the incision depth.
(5) Based on the statistical analysis of the test results, the calculation models for the fracture parameters of SFHSC with different strengths are obtained. The fracture toughness calculation model is expressed as $K_{\mathrm{sIC}}=\left(1+\alpha_{sf,k}\lambda_{f}^{2}\right)K_{\mathrm{IC}}$.The fracture energy calculation model is expressed as $G_{sF}=\left(1+\alpha_{sf,g}\lambda_{f}^{2}\right)G_{F}$.

In this paper, the fracture behavior of SHFSC is only studied through TPBTs, which can be further verified by comparative tests. The research on SHFSC materials is developing rapidly. Future work should explore the bridging role of fibers at a deeper level. The research will use concrete tests and construct a micro finite element model. Future work will also propose a model suitable for concrete design and application. Finally, a model suitable for concrete design and application will also be proposed.

## Figures and Tables

**Table 1:** Material properties of the steel fiber types.

Steel Fiber Type	Average Length l_f_/mm	Equivalent Diameter d_f_/mm	Aspect Ratio l_f_/d_f_
Milled steel fiber	31.312	0.9936	31.51
Bow steel fiber	32.426	0.5648	57.40
Shear corrugated steel fiber	30.529	0.5592	54.60

**Table 2:** Mix proportions of SFHSC and HSC (unit, kg/m^3^).

Mix No.	Cement	Water	W/C Ratio	Fine Aggregate	Coarse Aggregate	Superplasticizer (FDN-1)	*ρ_f_*
MF00	487	146	0.3	618	1199	4.87	0.0
MFT05-0	520	156	0.3	710	1065	5.20	0.0
MFT05	520	156	0.3	710	1065	5.20	39.3
MFT10-0	547	164	0.3	696	1044	5.47	0.0
MFT10	547	164	0.3	696	1044	5.47	78.6
MFT15-0	573	172	0.3	682	1023	5.73	0.0
MFT15	573	172	0.3	682	1023	5.73	117.9
MFT20-0	600	180	0.3	668	1002	6.00	0.0
MFT20	600	180	0.3	668	1002	6.00	157.2
MFT25-0	627	188	0.3	654	981	6.27	0.0
MFT25	627	188	0.3	654	981	6.27	196.5
BFT15-0	573	172	0.3	682	1023	5.73	0.0
BFT15	573	172	0.3	682	1023	5.73	117.9
SFT15-0	573	172	0.3	682	1023	5.73	0.0
SFT15	573	172	0.3	682	1023	5.73	117.9

Note: The first two letters indicate the type of steel fiber. T indicates that the specimen is a three-point bending specimen. -0 indicates the SHFSC comparison test specimen. MFT00 represents the reference group HSC.

**Table 3:** TPBT results for fracture properties of SFHSC and HSC specimens.

Mix No.	*ρ_f_*/%	a/h_0_	Peak Load P_max_/kN	Subcritical Crack Growth Length Δa/mm	K_IC_/MPa·m^1/2^	G_F_/N·m^−1^	CMOD_C_/mm	CTOD_C_/mm
MFT00-4	0.0	0.4	3.1555	34.25	2.0679	117.9977	0.0903	0.0343
MFT00-5	0.0	0.5	2.2942	23.34	1.8981	116.0086	0.0852	0.0291
MFT05-4	0.5	0.4	3.1915	31.68	2.2890	290.7975	0.0980	0.0328
MFT05-5	0.5	0.5	1.7311	30.73	1.7531	339.9776	0.1039	0.0271
MFT05-0-4	0.0	0.4	3.4333	20.97	1.7222	122.8843	0.0609	0.0212
MFT05-0-5	0.0	0.5	*	*	*	*	*	*
MFT10-4	1.0	0.4	3.6135	30.98	2.6288	614.5192	0.1184	0.0372
MFT10-5	1.0	0.5	2.8067	29.72	3.1170	914.7674	0.1731	0.0834
MFT10-0-4	0.0	0.4	2.6569	25.01	1.7615	97.4028	0.0639	0.0223
MFT10-0-5	0.0	0.5	1.4566	29.50	1.6891	86.2186	0.0887	0.0332
MFT15-4	1.5	0.4	3.9438	33.53	4.8897	1023.5237	0.2053	0.0736
MFT15-5	1.5	0.5	3.2958	31.84	4.0708	1260.7336	0.2885	0.0976
MFT15-0-4	0.0	0.4	3.4175	31.72	1.7726	104.6507	0.0628	0.0290
MFT15-0-5	0.0	0.5	2.1774	26.63	1.8625	133.7628	0.0849	0.0265
MFT20-4	2.0	0.4	4.7587	12.56	6.2200	1924.8270	0.3106	0.1054
MFT20-5	2.0	0.5	3.3240	37.52	5.4453	1365.6888	0.3413	0.1199
MFT20-0-4	0.0	0.4	3.3998	24.80	1.8742	157.4831	0.0683	0.0216
MFT20-0-5	0.0	0.5	2.5554	23.77	1.7792	135.7786	0.0888	0.0269
MFT25-4	2.5	0.4	6.3708	43.43	8.3826	2842.1893	0.4394	0.1552
MFT25-5	2.5	0.5	5.0915	40.35	7.6635	2409.6982	0.5298	0.1833
MFT25-0-4	0.0	0.4	2.9207	23.38	1.7326	118.0064	0.0680	0.0289
MFT25-0-5	0.0	0.5	2.2196	25.03	1.7517	95.2540	0.0820	0.0253
BFT15-4	1.5	0.4	9.7843	61.05	29.5033	11,217.1258	2.5063	0.8449
BFT15-0-4	0.0	0.4	3.2905	25.37	2.0203	120.6065	0.0719	0.0239
SFT15-4	1.5	0.4	4.0623	31.99	3.7856	1825.1208	0.2512	0.0480
SFT15-0-4	0.0	0.4	3.0870	27.35	2.1178	117.0248	0.0803	0.0250

Note: The first two letters indicate the type of steel fiber.-4 and -5 indicate the relative incision depths are 0.4 and 0.5, respectively. T indicates that the specimen is a three-point bending specimen. * Indicates the variation in the test data. -0 indicates the SHFSC comparison test specimen. MFT00 represents the reference group HSC.

## Data Availability

Data sharing not applicable.
